# Poromicromechanics reveals that physiological bone strains induce osteocyte-stimulating lacunar pressure

**DOI:** 10.1007/s10237-015-0704-y

**Published:** 2015-07-30

**Authors:** Stefan Scheiner, Peter Pivonka, Christian Hellmich

**Affiliations:** Institute for Mechanics of Materials and Structures, TU Wien—Vienna University of Technology, Karlsplatz 13/202, A-1040 Vienna, Austria; St. Vincent’s Department of Surgery, The University of Melbourne, Clinical Science Building, 29 Regent Street, VIC, 3065 Australia

**Keywords:** Poroelasticity, Micromechanics, Osteocytes, Bone remodeling, Mechanosensing, Hydrostatic pressure

## Abstract

Mechanical loads which are macroscopically acting onto bony organs, are known to influence the activities of biological cells located in the pore spaces of bone, in particular so the signaling and production processes mediated by osteocytes. The exact mechanisms by which osteocytes are actually able to “feel” the mechanical loading and changes thereof, has been the subject of numerous studies, and, while several hypotheses have been brought forth over time, this topic has remained a matter of debate. Relaxation times reported in a recent experimental study of Gardinier et al. (Bone 46(4):1075–1081, 2010) strongly suggest that the lacunar pores are likely to experience, during typical physiological load cycles, not only fluid transport, but also undrained conditions. The latter entail the buildup of lacunar pore pressures, which we here quantify by means of a thorough multiscale modeling approach. In particular, the proposed model is based on classical poroelasticity theory, and able to account for multiple pore spaces. First, the model reveals distinct nonlinear dependencies of the resulting lacunar (and vascular) pore pressures on the underlying bone composition, highlighting the importance of a rigorous multiscale approach for appropriate computation of the aforementioned pore pressures. Then, the derived equations are evaluated for macroscopic (uniaxial as well as hydrostatic) mechanical loading of physiological magnitude. The resulting model-predicted pore pressures agree very well with the pressures that have been revealed, by means of in vitro studies, to be of adequate magnitude for modulating the responses of biological cells, including osteocytes. This underlines that osteocytes may respond to many types of loading stimuli at the same time, in particular so to fluid flow and hydrostatic pressure.

## Introduction

Quite recently, Gardinier et al. (2010) presented a brilliant modification of the seminal work of Qin et al. (2002), allowing for the first time ever direct experimental access to the permeability of the lacunar-canalicular system of bone—they reported pressurization and relaxation times of around 8 s, relating to filling or drainage across the osteonal thickness, typically measuring about 65 microns (Gardinier et al. 2010). The underlying pressure intensification and relaxation system is governed by a constant pressure diffusion coefficient *c* (Zeng et al. 1994; Cowin 1999; Gardinier et al. 2010), being equal to the square of the characteristic length (here the osteonal thickness) over the characteristic relaxation time, $c = l^2/T_\text {relax} = 65^2 / 8 \approx 530\,{\upmu }\text {m}^2/\text {s}$. Accordingly, pressure relaxation through fluid flow over the distance between two lacunae, amounting to some 20 microns—see, e.g., Gardinier et al. (2010), Fig. 3b—takes about $T_\text {relax}=20^2/c\approx 0.8\,$s. In other words, for characteristic loading times of 1 s or more, canalicular fluid flow between lacunae is probable to occur, while the fluid is virtually trapped once loading times much below 1 s are encountered. Interestingly, both time regimes may be encountered under normal physiological loading, as can readily be derived from the wealth of experimental data available in the literature: Typical loading rates experienced in bone in vivo are accessible via force or strain measurements, see, e.g., Lanyon et al. (1975) and Bergmann et al. (1993). Magnitude and rate of these physical quantities allow for determination of characteristic loading times defined along the lines of Auriault et al. (2009),

1 $$\begin{aligned} T_\text {load}= \frac{|Q|}{|{\dot{Q}}|} \end{aligned}$$

**Fig. 1:**
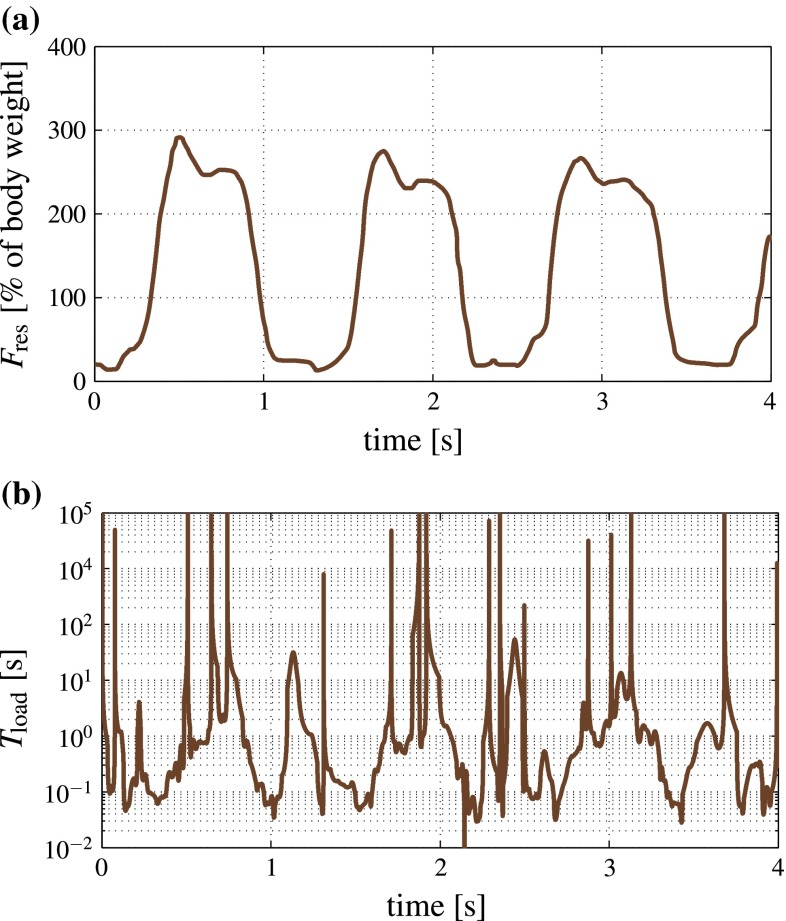
Bone loading experienced in the hip joint during walking on a treadmill at 2 km/h: **a** resultant force history as recorded by Bergmann et al. (1993), and **b** corresponding loading times $T_\text {load}$ according to Eq. (1)

with *Q* standing for the measured physical quantity, be it strain or force. During a typical load cycle experienced in vivo or subjected to in an in vitro experiment, the value for $T_\text {load}$ according to Eq. (1) obviously changes with time: It reaches infinity whenever the measured quantity reaches a maximum or minimum, and minimal characteristic times are encountered at time points in-between, typically close to the time instants at which the measured quantity exhibits maximum rates, see, e.g., Fig. 1a for the resultant force history associated to a human hip joint during walking (Bergmann et al. 1993). The occurring loading times illustrated in Fig. 1b, as derived from the force history of Fig. 1a by means of Eq. (1), may be binned into four time intervals characterizing different orders of magnitudes: load regime I with $T_\text {load} < 0.1$ s; load regime II with $0.1\,\text {s}\le T_\text {load}<1\,$s; load regime III with $1\,\text {s}\le T_\text {load}<10$ s; and load regime IV with $T_\text {load}\ge 10$ s, see Table 1. It appears that activities like uphill running, jogging, or walking imply a large fraction of characteristic loading times below a tenth of a second; this fraction may amount even up to 74 %, according to Mikić and Carter (1995), see Table 1. By contrast, static exercise regimes, such as knee bending (in sports medicine also known as “squats”) or the handling with dumbbells entail typically much longer characteristic loading times; e.g., almost 50 % of the slow movement of dumbbells from the lateral position to the front position and back, as recorded by Rohlmann et al. (2014), related to loading times larger than 10 s.

**Table 1:** Distribution of characteristic loading times related to in vivo mechanical loading data recorded in humans, for various organs and loading regimes; I: $T_\text {load}<0.1\,$s, II: $0.1\,\text {s}\le T_\text {load}< 1\,$s, III: $1\,\text {s}\le T_\text {load}< 10\,$s, and IV: $10\,\text {s}\le T_\text {load}$

References	Measured quantity	Loading regime	I (%)	II (%)	III (%)	IV (%)
Bergmann et al. (1993)	Hip joint force	Walking on a treadmill at 2 km/h	20	45	25	10
		Jogging on a treadmill at 8 km/h	39	51	6	4
		Stumbling without falling	4	62	28	6
Mikić and Carter (1995)	Anteromedial tibial strain energy	Walking on a treadmill at 1.4 m/s	56	36	6	2
		Walking on the floor with 71 kg additional weight	48	48	3	1
		Jogging on a treadmill at 2.2 m/s	74	22	3	1
Burr et al. (1996)	Tibial midshaft strains	Walking on the floor at 5 km/h	35	48	15	2
		Jogging on the floor at 10 km/h	55	33	9	3
		Zigzag-running uphill	58	33	5	4
Nikoyaan et al. (2009)	Shoulder joint force	Full range of shoulder motions	$\approx$0	30	57	13
Kutzner et al. (2010)	Knee joint force	Knee bending	$\approx$0	17	63	20
		Standing up and sitting down	3	35	43	19
		Ascending stairs	12	67	17	4
Rohlmann et al. (2014)	Spinal force	5 kg dumbbells moved from lateral to front position and back	0	1	51	48

All the loading times of Table 1, which amount to, or even exceed the 8 s-relaxation time suggested by Gardinier et al. (2010) for the osteonal scale, clearly indicate the occurrence of fluid flow through the canaliculi, as has been discussed and reported very extensively in the literature (Turner and Pavalko 1998; Qin et al. 2003; Knothe Tate 2003; Santos et al. 2009; Jacobs et al. 2010). On the other hand, a significant portion of the recorded loading times are well shorter than both the 8 s- and 0.8 s-relaxation times mentioned previously, so that undrained conditions are expected to occur during physiological loading as well, both at the osteonal and the inter-lacunar scale. Undrained conditions imply pressurization of the lacunar fluid, and this strongly motivates to carefully reconsider hydrostatic pressure as an important stimulus for osteocytes, thus re-energizing a quite old discussion. In fact, the idea of a hydrostatic pressure stimulus is often attributed to Thompson (1936), see, e.g., Bassett (1968). While Thompson’s suggestion was mainly founded on intuition and plausibility, in vitro studies repeatedly confirmed that bone cells (not only osteocytes, but also osteoblasts, osteoclasts, and their progenitors) indeed exhibit altered activities when subjected to hydrostatic pressure at frequencies of up to 1 Hz, exhibiting amplitudes of several tens (to hundreds) of kilopascals, see Table 2 for a related literature review. However, while the in vitro stimulation of bone cells by hydrostatic pressure seems to be a generally accepted fact, there seems to be some doubt on whether the hydrostatic pressures identified as mechanical stimuli in vitro are actually occurring in vivo. This doubt is exemplified by a quotation from the famous paper of Duncan and Turner (1995), reading *“hydrostatic pressure almost never occurs in mineralized bone”*. This statement is true and false at the same time, depending on the length scale considered. At the millimeter length scale of a piece of cortical or trabecular bone, it is of course true, since most of the bones are subjected to stress gradients and exhibit one or two dominant loading directions, making the occurrence of hydrostatic pressure (at the millimeter scale) indeed extremely improbable. The setting changes, however, at the tens-of-microns length scale of a single lacunar pore (and of the osteocyte it hosts), where the millimeter-sized gradients are not “seen” anymore, and which therefore could be well subjected to hydrostatic pressure (at the micron scale).

**Table 2:** Summary of experimental evidence regarding cell excitation by means of hydrostatic pressure application

References	Cell type	Pressure magnitude	Pressure frequency	Observed effects
Imamura et al. (1990)	Osteoblast-like MC3T3-E1 cells	0.5–2 atm	Static	Inhibition of osteoblast differentiation, promotion of osteoclast production (“optimum” pressure: 100 kPa), increased PGE synthesis
Ozawa et al. (1990)	Mouse osteoblast-like MC3T3-E1 cell	1 and 3 atm	Continuous	Decreased osteoblast numbers, increased PGE2 expression
Klein-Nulend et al. (1995)	Osteocytes from chicken calvariae	13 kPa	0.3 Hz (1 s loading, 2 s relaxation), for 24 h	Increase in prostaglandin release (can enhance bone formation)
Roelofsen et al. (1995)	Neonatal mouse calvarial cells	13 kPa	0.3 Hz	Stimulation of osteoblastic activity, stimulation of actin expression, AP activity
Brighton et al. (1996)	Calvarial bone cells from neonatal rats, exhibiting an osteoblast phenotype	17.2–69 kPa	1 Hz, for 10 cycles	increased proliferation, increased cytosolic calcium concentration
Vergne et al. (1996)	ROS 17/2.8 (rat osteoblast-like cells)	50–90 kPa	1 and 0.1 Hz, 20 min test duration	Increase in cell saturation density (for a frequency of 1 Hz), decreases alkaline phosphatase activity
Rubin et al. (1997)	Marrow cells from tibiae and femurs of C57BL/6 mice	1–2 atm	Static	Decreased osteoclast formation, decrease in mRNA coding for the membrane-bound form of MCSF
Nagatomi et al. (2001)	Osteoblasts from the calvaria of neonatal rats	10–40 kPa	0.25 or 1 Hz, 1 h daily	Elongated pressure decreases osteoblast proliferation, the same pressure stimulus causes different effect on different cells
Nagatomi et al. (2002)	Bone marrow cells (source of osteoclasts) from rat femurs	10–40 kPa	1 Hz (sinusoidal wave form), for 1 h per day	Reduced osteoclast differentiation and resorption activity, lower concentration of IL-1$\alpha$, down-regulation of mRNA expression for IL-1$\alpha$, IL-1$\beta$, and TNF-$\alpha$
Nagatomi et al. (2003)	Osteoblasts isolated from rat calvariae	10–40 kPa	1 Hz, for 1 h daily	Increased type-I collagen mRNA expression, increased amount of acid-soluble collagen, increased calcium concentration
Takai et al. (2004)	Primary osteoblasts obtained from trabecular bone cores taken from the epiphyses of metacarpal bones from 3- to 4-month-old calves	3 MPa	0.33 Hz (triangle wave form), for 1 h/day	Increased osteoblast function (only when osteocytes are present), increased osteocyte viability
Maul et al. (2007)	Bone marrow progenitor cells from rats	10–16 kPa	1 Hz	Enhanced proliferation
Gardinier et al. (2009)	MC3T3 osteoblast-like cells	0–68 kPa	0.5 Hz	Increased anabolic response, increase in ATP release, increased COX-2 levels
Liu et al. (2009)	Bone marrow stromal cells from tibiae and femurs of rats	10–36 kPa	0.25 Hz (sinusoidal wave form)	Increase in osteoblast activity-related transcription factors
Liu et al. (2010)	MLO-Y4 osteocyte-like cells (i.e., deriving from cells extracted from transgenic mice)	68 kPa	0.5 Hz (triangular wave form), for 1 or 2 h	Decreased osteocyte apoptosis, increase in intracellular calcium (after 40 s, may be related to osteoblast activity), of RANKL/OPG ratio (after 2 h), and of COX-2 mRNA level (after 1 h)
Rottmar et al. (2011)	Human bone- derived cells, from the hip marrow	1–11 kPa	30 min stimulation, 7 h, 30 min break	Increased osteogenic differentiation and proliferation
Henstock et al. (2013)	Cells contained in whole femurs of chick foetuses	0–279 kPa	0.0001–2 Hz	Increased volume of diaphysial collar

The latter suggestion of course deserves further scrutiny, and while direct pressure measurements at the micron scale remain out of reach, significant progress in theoretical and computational bone micromechanics over the last 15 years makes it nowadays possible to indeed “downscale” macroscopic strains occurring in vivo, to the fluid pressures arising in the lacunar pore spaces of cortical or trabecular bone tissue, and to check whether the resulting pore pressures agree with those needed to stimulate the cells occurring in bone in vitro. This is exactly the scope of the present paper, the remainder of which is organized as follows:

After a review of the differently sized pore spaces found in bone (see Sect. 2.1), the fundamentals of poromicromechanics are shortly summarized, focussing thereby on the representation of the double-porous system consisting of vascular and lacunar pores with solid bone matrix in-between (see Sects. 2.2 and 2.3); and on the underlying multiscale homogenization scheme which was experimentally validated by tests on bones stemming from the entire vertebrate kingdom, for various physical properties, such as elasticity, wave propagation phenomena, viscoelasticity, and strength (see Sects. 2.4 and 2.5). This scheme then allows for computation of lacunar pressures under different physiologically relevant macroscopic loading scenarios, always involving undrained conditions in the lacunar pores as discussed earlier, but alternatively assuming drained vascular pores [as often expected under normal physiological loading (Smit et al. 2002)], or undrained vascular pores [as expected under traumatic conditions (Hellmich and Ulm 2005a; Bryant 1983)]. The corresponding pressure predictions are then compared to those which have been experimentally shown to stimulate a variety of biological cells in vitro (see Sect. 3.1). After a numerical study concerning age-related changes in bone (see Sect. 3.2), the paper is concluded by an extensive discussion, covering limitations as well as possible future extensions of the poromicromechanics model presented in this paper, as well as its relation to bone mechanobiology and various associated transport processes taking place in the lacunar-canalicular pore channel network (see Sect. 4).

## Poromicroelasticity of bone

The scientific discipline of poromechanics originally emerged from the industrial field of geoengineering (von Terzaghi 1923), where it still drives technological improvements through reliable, often closed form, solutions (Abousleiman et al. 1996), ever extending the seminal work of Biot (1941). Being, however, applicable to any porous medium, poromechanics has gained, during recent decades, increasing popularity in the biological field, with applications concerning cartilage (Huyghe et al. 2007; Hoang and Abousleiman 2009), brain (Mehrabian and Abousleiman 2011), wood (Bader et al. 2011), or bone (Cowin 1999). Concerning the latter, the theory of poromicromechanics (Dormieux et al. 2006), where not only porosities, but also additional microstructural features are explicitly considered for determining the mechanical interactions between pore pressures and stresses acting on porous material volumes, has been particularly successfully applied to bone (Hellmich and Ulm 2005a, b; Morin and Hellmich 2014), and in this context, has allowed for explaining various experimentally observed pore pressure buildup phenomena (Bryant 1983, 1988; McCarthy et al. 1990; Hosokawa and Otani 1997; Lee et al. 2003). Corresponding experimentally validated mathematical models are employed hereafter, in order to determine the lacunar and vascular pore pressures arising from physiological strains, preceded by a short review on the pore spaces found in bone, as described next.

### Pore spaces in bone

**Fig. 2:**
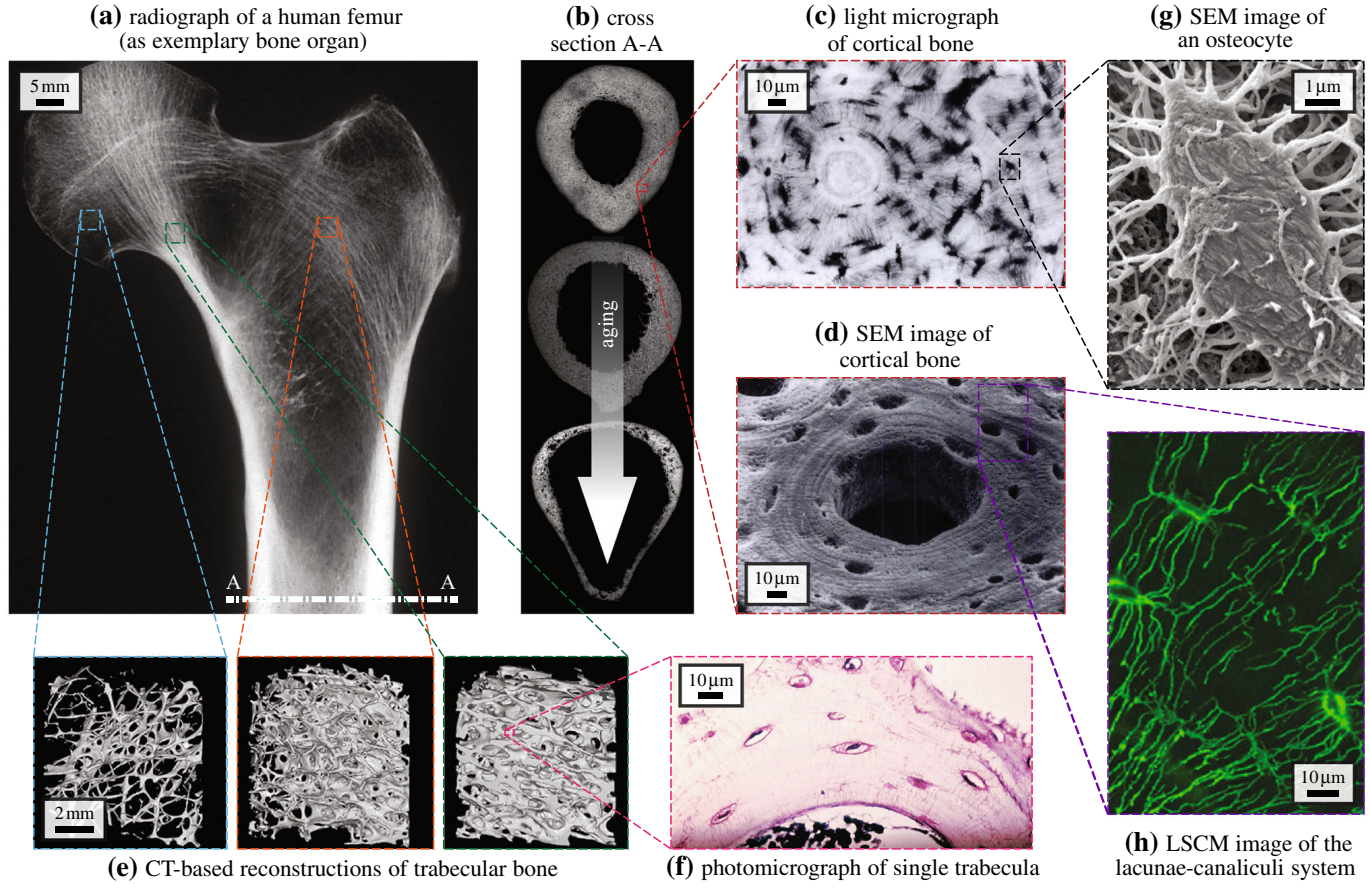
Hierarchical organization of bone relevant for bone remodeling–modulating pore pressures, presented by the example of the human femur: **a** X-ray image of the proximal part of a human femur, reproduced from Sinclair et al. (2013), with permission from Elsevier B.V.; **b** midshaft cross section A-A, illustrated through corresponding microradiographs of femur cross sections, by courtesy of John G. Clement and David Thomas (taken from the Melbourne Femur Collection), shows deteriorating integrity with increasing age; cortical bone microstructure and its main constituents acquired by means of **c** light microscopy, reprinted from Buckwalter and Cooper (1987), with permission from the American Academy of Orthopaedic Surgeons (AAOS), or **d** scanning electron microscopy (SEM), reprinted from Kessel and Kardon (1979), by courtesy of Randy H. Kardon; **e** shows computed tomography (CT) images of trabecular bone acquired at different locations showing different porosities, reproduced from Padilla et al. (2008), with permission from Elsevier B.V.; **f** a photomicrograph of a single trabecula shows the composition of trabecular bone, reproduced from Sinclair et al. (2013), with permission from Elsevier B.V.; **g** SEM allows to visualize the osteocytes residing in the lacunar pores detectable in cortical bone and trabecular bone, reprinted from Pajevic (2009), by permission from Macmillan Publishers Ltd. on behalf of Cancer Research UK: IBMS BoneKey,  2009; **h** laser scanning confocal microscopy (LSCM) shows the canaliculi connecting the lacunae and the therein residing osteocytes, forming a dense network embedded in the extracellular bone matrix, reproduced from Ebacher et al. (2012), with permission from Elsevier B.V.

The largest pores found in bone host blood vessels and are therefore often called vascular pores. In cortical bone (forming shell-type structures at the surface of whole bony organs), the vascular pores form a branching structure (Cooper et al. 2003), with the main branches (normally following the main anatomical directions of the organ) often being called Haversian canals, while the sideways to smaller branches are sometimes called Volkmann canals. Over the lifespan of an individual, the vascular porosity typically increases, from a few percent in young adults, up to 35 % and more at age 90 (Cooper et al. 2007), see Fig. 2a, b. Trabecular bone, surrounded by a cortical shell, can be found at the ends of long bones, proximal to joints, resulting from a perforation process of the cartilage originally laid down during the development of the biological individual (Buckwalter et al. 1995a, b; Byers et al. 2000), with vascular porosities (then also called inter-trabecular porosities) ranging from typically 50–90 % (Padilla et al. 2008; Boutroy et al. 2005, 2011), and also shows a great spatial variability within one and the same organ, see Fig. 2a, e. The interpenetration of vascular pores in trabecular bone results in the appearance of the extravascular bone matrix in-between, as struts or plates, which are called trabeculae. With aging, the latter undergo considerable thinning and may even be lost (Thomsen et al. 2002; Chen et al. 2010).

Within the extravascular bone matrix, another class of pores can be found, with characteristic sizes of ten micrometers, hosting the probably mechanosensitive osteocytes. The corresponding porosity can be straightforwardly determined from sufficiently high-resolution light or transmission/scanning electron micrographs (Buckwalter et al. 1995a; Tai et al. 2008), see Fig. 2c, d, f, and g; it amounts to about 10 % (of the space without the vascular pores). With aging, gradual apoptosis of the osteocytes is observed (Busse et al. 2010), allowing for mineralization of the lacunar spaces; thereby reducing the lacunar porosity.

The lacunae are connected by much smaller channels called canaliculi, which host the osteocyte processes connecting the osteocytes to a network similar to those made up by neurons. These channels have a characteristic diameter of a few 100 nm, typically around 500 nm (Reilly et al. 2001; Sharma et al. 2012), see Fig. 2h. Since these canalicular pores are much smaller than the lacunar pores, we regard them throughout this paper as a porosity which is of course physically linked to, but at the same time “length-scale-separated” from the lacunar pores. Namely, length-scale separation is one of the most fundamental and governing principles in the very old and therefore highly mature scientific field of continuum mechanics (Salençon 2001), and its subfields micromechanics (Zaoui 2002) and poromechanics (Coussy 2004; Dormieux et al. 2006), which will be used hereafter to elucidate the mechanical functioning of the different pore spaces found in bone material. Given our focus on the osteocytes and their mechanical environment, all pore spaces smaller than the lacunar pore space, such as the canalicular pore space at the 100 nanometers scale, but also the inter-crystalline and inter-molecular pore spaces at the ten nanometers and the single nanometer scales (Hellmich et al. 2009) will not be explicitly introduced here, but they are considered as an integral part of what we will call in the following “extralacunar bone matrix,” i.e., extracellular bone matrix *plus* canaliculi.

### Separation of scales—representative volume elements

In the following, the mechanical impact of the aforementioned pore spaces is studied within the framework of continuum micromechanics (Hill 1963, 1965; Suquet 1997; Zaoui 1997, 2002; Dormieux et al. 2006), where a material is understood as a macrohomogeneous, but microheterogeneous body filling a representative volume element (RVE) with characteristic length $\ell _\text {RVE}$, fulfilling the following separation-of-scales conditions: (i) $\ell _\text {RVE} \gg d_\text {RVE}$, $d_\text {RVE}$ representing the characteristic length of inhomogeneities within the RVE, and (ii) $\ell _\text {RVE} \ll \{{\mathscr {L}},{\mathscr {P}}\}$, ${\mathscr {L}}$ representing the characteristic length of the geometry and $\mathscr {P}$ representing the characteristic length of the loading of a structure built up by the material defined on the RVE.

In general, the microstructure within one RVE is so complicated that it cannot be described in complete detail. Therefore, quasi-homogeneous subdomains with known physical properties are reasonably chosen. They are called material phases, typically comprising solid and pore phases. The homogenized (upscaled) poroelastic behavior of the material on the observation scale of the RVE, i.e., the relation between homogeneous deformations acting on the boundary of the RVE, the pressures acting inside the pores, and the resulting macroscopic (average) stresses, can then be estimated from the elastic behavior of the material phases, their volume fractions within the RVE, their characteristic shapes, and their interactions. If a single phase exhibits a heterogeneous microstructure itself, its mechanical behavior can be estimated by introduction of an RVE within this phase, with dimensions $\ell _{\text {RVE},2}\le d_\text {RVE}$, comprising again smaller phases with characteristic length $d_{\text {RVE},2}\ll \ell _{\text {RVE},2}$, and so on. This leads to a multistep homogenization scheme.

**Fig. 3:**
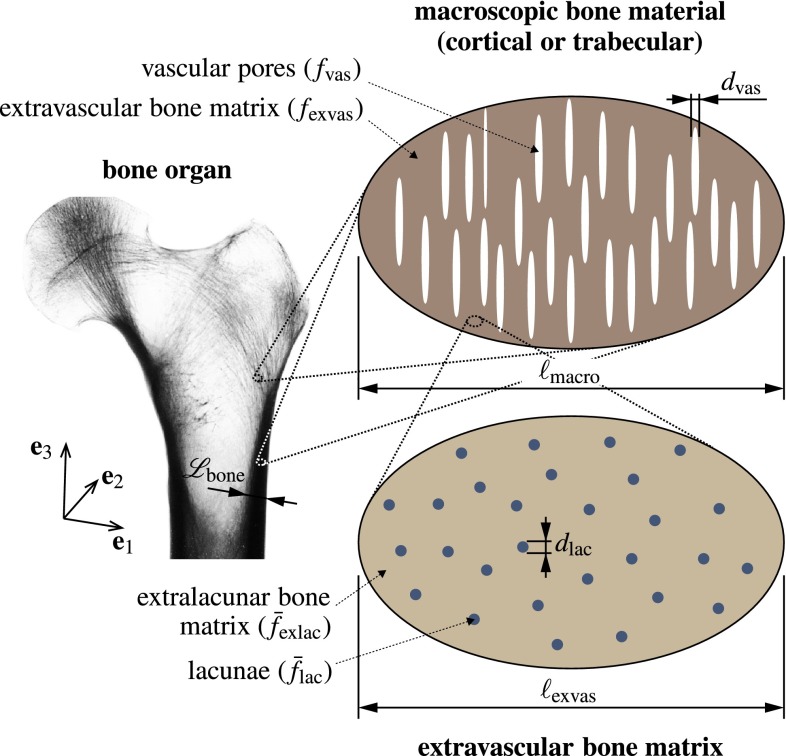
Micromechanical representation of cortical bone, based on which the poromicromechanical model is developed: Cortical bone microstructure is composed of extravascular bone matrix, with volume fraction $f_\text {exvas}$, and vascular pore space, with volume fraction $f_\text {vas}$, $f_\text {exvas}+f_\text {vas}=1$, $\mathscr {L}_\text {bone}\gg \ell _\text {macro}\gg d_\text {vas}$, whereas extravascular bone matrix is composed of extracellular bone matrix, with volume fraction $\bar{f}_\text {exlac}$, and lacunar pores, with volume fraction $\bar{f}_\text {lac}$, $\bar{f}_\text {exlac}+\bar{f}_\text {lac}=1$, $\ell _\text {exvas}\gg d_\text {lac}$; the X-ray image of the bone organ was reproduced from Sinclair et al. (2013), with permission of Elsevier B.V.

In the case of bone, we adopt a homogenization scheme which has been extensively validated against experimental data across a multitude of bone samples harvested from different anatomical locations of different species of different ages and various physical quantities such as elasticity (Hellmich et al. 2004; Fritsch and Hellmich 2007), poroelasticity (Hellmich and Ulm 2005a, b; Morin and Hellmich 2014), viscoelastcity (Eberhardsteiner et al. 2014), and elastoplasticity (strength) (Fritsch et al. 2009). In the course of this adoption, we start with zooming out of a bony organ with a characteristic length of several mm to cm, $\mathscr {L}_\text {bone}=5...10\times 10^{-3}\,$m, an RVE of (cortical or trabecular) bone, see Fig. 3, which in the case of cortical bone exhibits a typical length of $\ell _\text {macro}\approx 1...5\times 10^{-3}\,$m (Lees et al. 1979; Padilla et al. 2008), and being somewhat larger in the case of trabecular bone. Within this RVE, we distinguish the phases “vascular pores,” exhibiting a characteristic size of $d_\text {vas}=50...80\times 10^{-6}$ m and cylindrical shape, and “extravascular bone matrix,” whereby the former are embedded into the latter (Hellmich et al. 2004, 2008; Yosibash et al. 2008; Fritsch et al. 2009; Grimal et al. 2011; Colloca et al. 2014), see Fig. 3. When zooming into a piece of the extravascular bone matrix, it appears as a porous material itself, represented by means of an RVE with a characteristic length of $\ell _\text {exvas}\approx 100...200\times 10^{-6}\,$m, being composed of the phases “extralacunar bone matrix” (i.e., extracellular bone matrix plus the therein embedded, comparatively tiny canalicular channels) and “lacunar pores” with characteristic size $d_\text {lac}\approx 10\times 10^{-6}\,$m (Buckwalter et al. 1995a; Martin et al. 1998), approximated by spherical inclusions (Fritsch and Hellmich 2007; Hellmich et al. 2009; Morin and Hellmich 2014). We note in passing that $d_\text {RVE}$ and $\ell _\text {RVE}$ are always separated by more than a factor of three, which already allows homogenization results with a low error, of not more than around 3 % (Drugan and Willis 1996).

### Micro–macro relations in the double-porous medium

In the framework of linearized (small) strains (standardly used in the mechanical study of mineralized tissues) and elastic behavior of the solid phases of the RVEs depicted in Fig. 3, the “macroscopic” stresses $\varvec{\varSigma }$ related to the macroscopic (cortical or trabecular) RVE (i.e., the spatial average of microstresses within this RVE) are linearly related to the “macroscopic” strains $\mathbf {E}$ imposed as displacements at the boundary of this RVE, and to the pore pressures $p_\text {lac}$ and $p_\text {vas}$ acting inside the vascular and lacunar pores, respectively,

2 $$\begin{aligned} \varvec{\varSigma }= \mathbb {C}_\text {macro}:\mathbf {E}- \mathbf {b}_\text {macro}^\text {lac}p_\text {lac}- \mathbf {b}_\text {macro}^\text {vas}p_\text {vas}\,, \end{aligned}$$

as has been derived in theoretical detail in Dormieux et al. (2006), Hellmich et al. (2009) and Pichler and Hellmich (2010). This multilinearity is expressed by the “drained” cortical stiffness tensor $\mathbb {C}_\text {macro}$ (related to drained lacunar and vascular pores, meaning that the pore pressures are governed by effects from outside the RVE, and comprising particularly the special case of “zero pressures”), and by the pore space-specific Biot tensors $\mathbf {b}_\text {macro}^\text {vas}$ and $\mathbf {b}_\text {macro}^\text {lac}$, which quantify the macroscopic stresses $\varvec{\varSigma }$ arising in an undeformed cortical RVE, from pressures acting in the two considered pore spaces. Mathematical details on how the second-order Biot tensors are derived from the micromechanical representation depicted in Fig. 3, are given in the Appendix of this paper.

The macroscopic strains and the pore pressures also lead to changes in the lacunar and vascular porosities, again in a multilinear way,

3 $$\begin{aligned} f_\text {lac}-f_{\text {lac},0}= \mathbf {b}_\text {macro}^\text {lac}:\mathbf {E}+ \frac{p_\text {lac}}{N_\text {macro}^\text {lac,lac}}+ \frac{p_\text {vas}}{N_\text {macro}^\text {lac,vas}} \end{aligned}$$

and

4 $$\begin{aligned} f_\text {vas}-f_{\text {vas},0}= \mathbf {b}_\text {macro}^\text {vas}:\mathbf {E}+ \frac{p_\text {lac}}{N_\text {macro}^\text {vas,lac}}+ \frac{p_\text {vas}}{N_\text {macro}^\text {vas,vas}}\,, \end{aligned}$$

where $f_{\text {lac},0}$ and $f_{\text {vas},0}$ are the initial volume fractions of lacunar and vascular pore spaces (quantified on the macroscopic observation scale), before the RVE of macroscopic (cortical or trabecular) bone is subjected to mechanical loading. Furthermore, $N_\text {macro}^{j,j}$ and $N_\text {macro}^{j,k}=N_\text {macro}^{k,j}$ are the so-called Biot moduli, whose nomenclature is built as follows: Biot modulus $N_r^{j,k}$ considers the effect of the pressure in pore space *k* on the porosity change of pore space *j*, whereby both pore spaces are measured in RVE *r*. We note that the theoretical derivation of Eqs. (3) and (4) is described in greater detail elsewhere (Dormieux et al. 2006; Hellmich et al. 2009; Pichler and Hellmich 2010), and that the Biot moduli are functions of the Biot tensors and of the extralacunar stiffness tensor, as given in more detail in the Appendix of this paper.

### Sealing of pore spaces I—undrained lacunar pores

So far, the pore pressures $p_\text {lac}$ and $p_\text {vas}$ were considered as independent loading variables, governed by the fluid flow conditions met in the double-porous medium. We will now investigate specific cases of these fluid flow conditions. The first case relates to the situation where the macroscopic strains $\mathbf {E}$ (and the corresponding strains acting on the lacunar pores) are built up so quickly that the pore fluid cannot leave any more the lacunar pore space, and that it is therefore “trapped” therein, see the first paragraph of Sect. 1, Table 1, and Fig. 1 for the corresponding physiological conditions. Under such conditions, the lacunar pore deformation is solely governed by the deformation of the lacunar fluid (also comprising the osteocyte) itself, see Hellmich and Ulm (2005a), Hellmich et al. (2009) and Coussy (2004) for further theoretical details,

5 $$\begin{aligned} \frac{f_\text {lac}-f_{\text {lac},0}}{f_{\text {lac},0}}= -\frac{p_\text {lac}}{k_\text {lac}} \end{aligned}$$

where $k_\text {lac}$ denotes the bulk modulus of the fluid contained in the lacunar pores, which is standardly approximated by that of water, $k_\text {lac} = 2.3\,$GPa (Murdock 1996). At the same time, we consider the case where the aforementioned loading is still slow enough so as to allow the vascular fluid to equilibrate with the (comparatively low) pressure stemming from blood circulation, usually ranging between 10 and 30 mmHg (i.e., 1.33–4 kPa) (Brookes and Revell 1998; Cameron et al. 1999), which we approximate as $p_\text {vas}\approx 0$. Insertion of Eq. (5) into Eq. (3) then leads to the following result:

6 $$\begin{aligned} p_\text {lac}= -M_\text {macro}^\text {lac}\mathbf {b}_\text {macro}^\text {lac}:\mathbf {E}\,, \end{aligned}$$

with the modulus-type quantity $M_\text {macro}^\text {lac}$ defined standardly as (Coussy 2004; Hellmich et al. 2009)

7 $$\begin{aligned} \dfrac{1}{M_\text {macro}^\text {lac}}= \frac{f_{\text {lac},0}}{k_\text {lac}}+\frac{1}{N_\text {macro}^{\text {lac},\text {lac}}}\,. \end{aligned}$$

Insertion of Eq. (6) and $p_\text {vas}=0$ into Eq. (2) yields a stress-strain relation of the format

8 $$\begin{aligned} \varvec{\varSigma }= \mathbb {C}_\text {macro}^\text {lac-u}:\mathbf {E}\,, \end{aligned}$$

with the stiffness tensor $\mathbb {C}_\text {macro}^\text {lac-u}$ referring to undrained lacunar pores and drained vascular pores,

9 $$\begin{aligned} \mathbb {C}_\text {macro}^\text {lac-u}= \mathbb {C}_\text {macro}+M_\text {macro}^\text {lac} \mathbf {b}_\text {macro}^\text {lac}\otimes \mathbf {b}_\text {macro}^\text {lac}\,. \end{aligned}$$

Insertion of Eq. (8) into Eq. (6) then gives

10 $$\begin{aligned} p_\text {lac} =-\left( \mathbf {B}^\text {lac}_\text {macro}\right) _\text {lac-u}:\varvec{\varSigma }\,, \end{aligned}$$

with the second-order Skempton tensor $\left( \mathbf {B}^\text {lac}_\text {macro}\right) _\text {lac-u}$ linearly relating macroscopic stresses to the lacunar pore pressure, reading as

11 $$\begin{aligned} \left( \mathbf {B}^\text {lac}_\text {macro}\right) _\text {lac-u}= M_\text {macro}^\text {lac}\mathbf {b}_\text {macro}^\text {lac}: \left( \mathbb {C}_\text {macro}^\text {lac-u}\right) ^{-1}. \end{aligned}$$

### Sealing of pore spaces II—undrained lacunar and vascular pores

Now we consider the case where also the vascular fluid is “trapped” in its pore space, due to sufficiently high loading rates, of typical characteristic times in the millisecond regime (Bryant 1983; Hellmich and Ulm 2005a). Then, also the vascular porosity changes are driven solely by the compression of the vascular fluid,

12 $$\begin{aligned} \frac{f_\text {vas}-f_{\text {vas},0}}{f_{\text {vas},0}}= -\frac{p_\text {vas}}{k_\text {vas}}\,, \end{aligned}$$

where we approximate also the vascular bulk modulus by that of water, $k_\text {vas}=2.3$ GPa (Murdock 1996). Insertion of Eqs. (5) and (12) into Eqs. (3) and (4) yields a linear system of equations for the two unknowns $p_\text {lac}$ and $p_\text {vas}$, with the solutions

13 $$\begin{aligned} \begin{aligned} p_\text {lac}=&\,- \frac{M_\text {macro}^\text {lac}N_\text {macro}^\text {lac,vas}}{(N_\text {macro}^\text {lac,vas})^2-M_\text {macro}^\text {lac} M_\text {macro}^\text {vas}}\\&\,\times \left( \mathbf {b}_\text {macro}^\text {vas} M_\text {macro}^\text {vas}+ \mathbf {b}_\text {macro}^\text {lac}N_\text {macro}^\text {lac,vas}\right) : \mathbf {E} \end{aligned} \end{aligned}$$

and

14 $$\begin{aligned} \begin{aligned} p_\text {vas}=&\,- \frac{M_\text {macro}^\text {vas}N_\text {macro}^\text {lac,vas}}{(N_\text {macro}^\text {lac,vas})^2-M_\text {macro}^\text {lac} M_\text {macro}^\text {vas}}\\&\,\times \left( \mathbf {b}_\text {macro}^\text {lac}M_\text {macro}^ \text {lac}+ \mathbf {b}_\text {macro}^\text {vas}N_\text {macro}^\text {lac,vas}\right) : \mathbf {E}\,, \end{aligned} \end{aligned}$$

whereby the modulus-type quantity $M_\text {macro}^\text {vas}$ again follows the standard definition (Coussy 2004)

15 $$\begin{aligned} \frac{1}{M_\text {macro}^\text {vas}}= \frac{f_{\text {vas},0}}{k_\text {vas}}+\frac{1}{N_\text {macro}^{\text {vas},\text {vas}}}\,. \end{aligned}$$

Insertion of Eqs. (13) and (14) into Eq. (2) yields a stress-strain relation in the format

16 $$\begin{aligned} \varvec{\varSigma }= \mathbb {C}_\text {macro}^\text {lac,vas-u}:\mathbf {E}\,, \end{aligned}$$

with the stiffness tensor $\mathbb {C}_\text {macro}^\text {lac,vas-u}$ referring to undrained lacunar and vascular pores,

17 $$\begin{aligned} \mathbb {C}^\text {lac,vas-u}_\text {macro}= & {} \mathbb {C}_\text {macro} + \mathbf {b}_\text {macro}^\text {lac}\otimes \Bigg [ \frac{M_\text {macro}^\text {lac}N_\text {macro}^\text {lac,vas}}{(N_\text {macro}^\text {lac,vas})^2-M_\text {macro}^\text {lac} M_\text {macro}^\text {vas}}\nonumber \\&\times \, \left( \mathbf {b}_\text {macro}^\text {vas}M_\text {macro}^\text {vas}+ \mathbf {b}_\text {macro}^\text {lac}N_\text {macro}^\text {lac,vas}\right) \Bigg ]\nonumber \\&+\,\mathbf {b}_\text {macro}^\text {vas}\otimes \Bigg [ \frac{M_\text {macro}^\text {vas}N_\text {macro}^\text {lac,vas}}{(N_\text {macro}^\text {lac,vas})^2-M_\text {macro}^\text {lac} M_\text {macro}^\text {vas}} \nonumber \\&\times \, \left( \mathbf {b}_\text {macro}^\text {lac}M_\text {macro}^\text {lac}+ \mathbf {b}_\text {macro}^\text {vas}N_\text {macro}^\text {lac,vas}\right) \Bigg ]. \end{aligned}$$

Insertion of Eq. (17) into Eqs. (13) and (14) then yields

18 $$\begin{aligned} p_\text {lac}= -\left( \mathbf {B}^\text {lac}_\text {macro}\right) _\text {lac,vas-u}:\varvec{\varSigma }\,, \end{aligned}$$

and

19 $$\begin{aligned} p_\text {vas}= -\left( \mathbf {B}^\text {vas}_\text {macro}\right) _\text {lac,vas-u}:\varvec{\varSigma }\,, \end{aligned}$$

with the second-order Skempton tensors $\left( \mathbf {B}^\text {lac}_\text {macro}\right) _\text {lac,vas-u}$ and $\left( \mathbf {B}^\text {vas}_\text {macro}\right) _\text {lac,vas-u}$ reading as

20 $$\begin{aligned} \left( \mathbf {B}_\text {macro}^\text {lac}\right) _\text {lac,vas-u}= & {} \frac{M_\text {macro}^\text {lac}N_\text {macro}^\text {lac,vas}}{(N_\text {macro}^\text {lac,vas})^2-M_\text {macro}^\text {lac} M_\text {macro}^\text {vas}}\nonumber \\&\,\times \left( \mathbf {b}_\text {macro}^\text {vas}M_\text {macro}^\text {vas}+ \mathbf {b}_\text {macro}^\text {lac}N_\text {macro}^\text {lac,vas}\right) \nonumber \\&\,:\left( \mathbb {C}_\text {macro}^\text {lac,vas-u}\right) ^{-1} \end{aligned}$$

and

21 $$\begin{aligned} \left( \mathbf {B}_\text {macro}^\text {vas}\right) _\text {lac,vas-u}= & {} \frac{M_\text {macro}^\text {vas}N_\text {macro}^\text {lac,vas}}{(N_\text {macro}^\text {lac,vas})^2-M_\text {macro}^\text {lac} M_\text {macro}^\text {vas}}\nonumber \\&\times \left( \mathbf {b}_\text {macro}^\text {lac}M_\text {macro}^\text {lac}+ \mathbf {b}_\text {macro}^\text {vas}N_\text {macro}^\text {lac,vas}\right) \nonumber \\&:\left( \mathbb {C}_\text {macro}^\text {lac,vas-u}\right) ^{-1}\,. \end{aligned}$$

## Results

### Pore pressure built up by macroscopic stress and strain states

Evaluation of Eqs. (2)–(21), together with Eqs. (23)–(38), reveals that the Skempton tensors given by Eqs. (11), (20), and (21) are of diagonal format. That is, in a base frame $\mathbf {e}_1$, $\mathbf {e}_2$, and $\mathbf {e}_3$ coinciding with the anatomical and pore directions, see Fig. 3, all components $B_{ij}$ with mixed indices, $i\ne j$ vanish, and only the “normal” components $B_{11}$, $B_{22}$, and $B_{33}$ remain. Hence, the pressure buildup can be given explicitly as

22 $$\begin{aligned} p = B_{11}\varSigma _{11} + B_{22}\varSigma _{22} + B_{33}\varSigma _{33} \end{aligned}$$

**Fig. 4:**
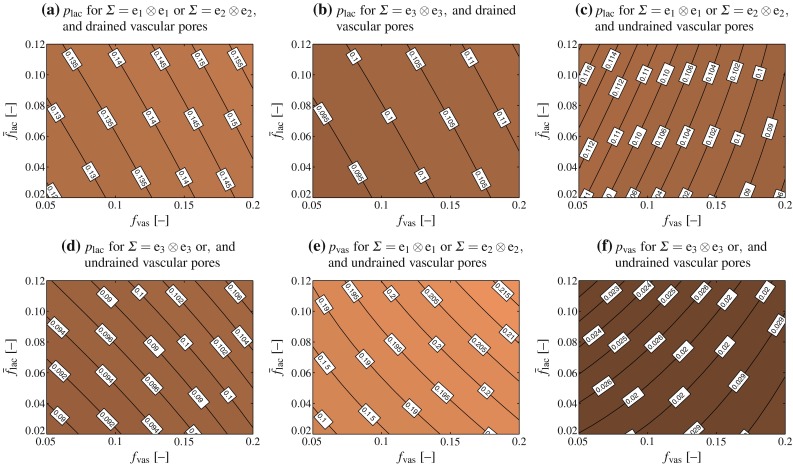
Lacunar (**a**–**d**) and vascular (**e**, **f**) pore pressures building up in response to uniaxial macroscopic unit stresses; the pore pressures shown in **a** and **b** follow from evaluation of Eq. (10), whereas the pore pressures shown in **c**–**f** follow from evaluation of Eqs. (18) and (19)

**Fig. 5:**
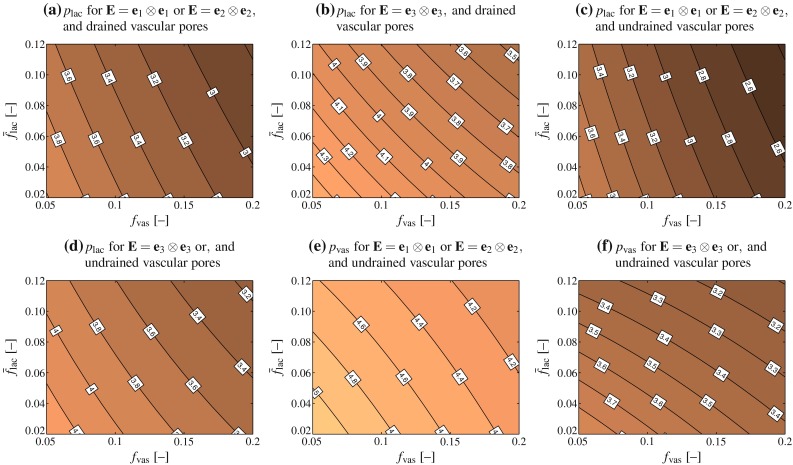
Lacunar (**a**–**d**) and vascular (**e**, **f**) pore pressures, in kPa, building up in response to uniaxial macroscopic unit microstrains; the pore pressures shown in **a** and **b** follow from evaluation of Eq. (6), whereas the pore pressures shown in **c**–**f** follow from evaluation of Eqs. (13) and (14)

We directly observe that macroscopic shear loading in the anatomical directions does not induce any pressure buildup. However, this does not mean that macroscopic shearing per se never builds up pore pressure: Whenever the orientation of the shear stress vectors would deviate from the anatomical directions, then they would induce corresponding normal stress tensor components with respect to the anatomical directions, and this would of course evoke pore pressures. What can be also seen from Eq. (22) is that the effect of macroscopic normal stresses in the anatomical directions, on the pore pressure buildup is (anatomical) direction-dependent. This is further quantified in Fig. 4. The lacunar and vascular pressure buildup is lower in axial than in transverse direction. Also, the lacunar pore pressure buildup for the case of drained vascular pores is expectedly higher than for undrained vascular pores. This increase is direction-dependent as well; while the increase is in the range of only a few percent for macroscopic stresses applied in the principal anatomical (axial) direction, it may reach more than 20 % in the transverse direction. Similar deliberations hold for the strain–pore pressure relations given by Eq. (13) and (14), see Fig. 5.

**Fig. 6:**
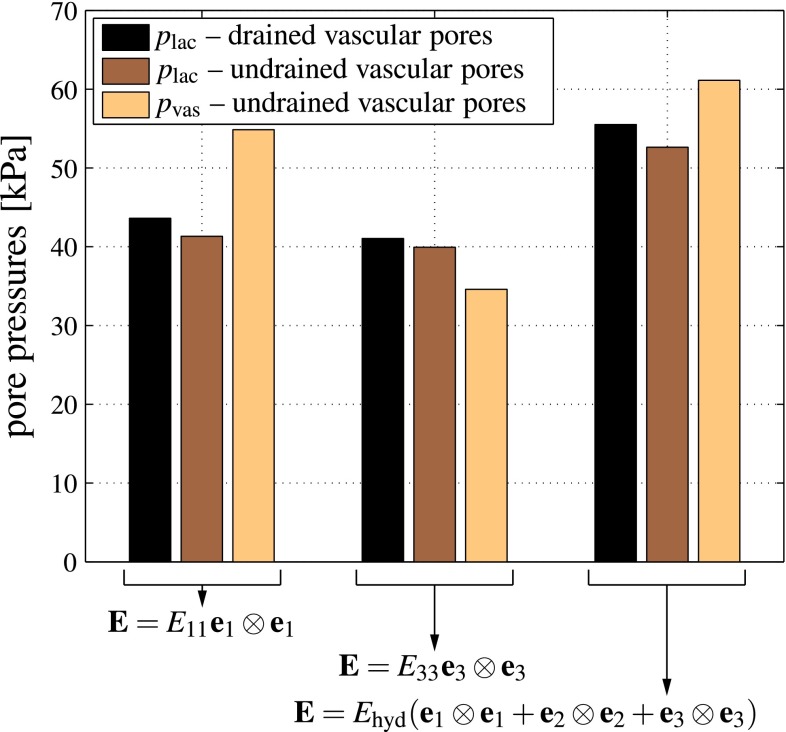
Lacunar and vascular pore pressures building up in correspondence to physiological macroscopic (uniaxial and hydrostatic) strains; strain magnitudes are chosen according the requirement of strain energy density equivalence

In order to study this direction dependence in more detail, we consider a typical healthy cortical bone, with porosities $\bar{f}_\text {lac}=0.1$ and $f_\text {vas}=0.05$ (Buckwalter et al. 1995a; Martin et al. 1998; Cooper et al. 2007; Tai et al. 2008). Furthermore, we aim at elucidating the effect of macroscopic physiological strains. Namely, considering in vivo strain measurements (Fritton et al. 2000; Lanyon et al. 1975), a (frequently occurring) macroscopic strain in the direction of the long axis of the bone, i.e., parallel to base vector $\mathbf {e}_3$, is prescribed, $E_{33}=-10\times 10^{-6}$ and $\mathbf {E}=E_{33}\mathbf {e}_3\otimes \mathbf {e}_3$. Our model predicts that for drained vascular pores, this loading causes a pressure buildup in the lacunar pores amounting to 41.05 kPa, while for undrained vascular pores the pressure that buildups in the lacunar and vascular pores, respectively, amounts to 39.93 and 34.59 kPa, respectively, see Fig. 6. On the level of the entire macroscopic RVE, this leads to a storage of elastic strain energy density amounting to

$$\begin{aligned} \varPsi ^\text {lac-u}=1.1326\,\text {Pa} \end{aligned}$$

in the case of undrained lacunar and drained vascular pores, and to

$$\begin{aligned} \varPsi ^\text {lac,vas-u}=1.1501\,\text {Pa} \end{aligned}$$

in the case of both lacunar and vascular pores being undrained, see Eqs. (40) and (43) for mathematical details. For storing the same amount of elastic energy in case of uniaxial transverse loading, $\mathbf {E}=E_{11}\mathbf {e}_1\otimes \mathbf {e}_1$, the latter strains need to be of magnitude

$$\begin{aligned} E_{11}^\text {lac-u}=-11.7359\times 10^{-6} \end{aligned}$$

in the case of undrained lacunar and drained vascular pores, and to

$$\begin{aligned} E_{11}^\text {lac,vas-u}=-11.5975\times 10^{-6} \end{aligned}$$

in the case of both lacunar and vascular pores being undrained, see Eqs. (40) and (43) for mathematical details. The corresponding lacunar pore pressures amount to 43.61 and 41.33 kPa, respectively (for drained and undrained vascular pores, respectively), while the corresponding vascular pore pressure for undrained vascular pores amounts to 54.84 kPa, see Fig. 6. Hence, the macrostrain-induced lacunar pore pressure is fairly independent of whether a certain amount of energy is transferred to a macroscopic RVE in terms of axial or transverse macroscopic strains. Next, we check the effect of subjecting the RVE again to the same amount of energy, but now in terms of hydrostatic macroscopic strain, $\mathbf {E}=E_\text {hyd}(\mathbf {e}_1\otimes \mathbf {e}_1+\mathbf {e}_2\otimes \mathbf {e}_2+\mathbf {e}_3\otimes \mathbf {e}_3)$. Accordingly, $E_\text {hyd}$ amounts to

$$\begin{aligned} E_\text {hyd}^\text {lac-u}=-4.8111\times 10^{-6} \end{aligned}$$

in the case of undrained lacunar and drained vascular pores, and to

$$\begin{aligned} E_\text {hyd}^\text {lac,vas-u}=-4.7319\times 10^{-6} \end{aligned}$$

in the case of both lacunar and vascular pores being undrained, see Eqs. (41) and (44) for mathematical details. The corresponding lacunar pressures amount to 55.51 and 52.62 kPa, respectively (for drained and undrained vascular pores, respectively), while the corresponding vascular pore pressure for undrained vascular pores amounts to 61.12 kPa, see Fig. 6. The differences between the extreme cases of purely uniaxial and fully hydrostatic loading applied on the macroscopic scale, in terms of their effect on the hydrostatic pressure buildup in the lacunar space, are not more than some 20 %—hence, the local hydrostatic pressure is fairly independent from the hydrostatic or non-hydrostatic nature of the macroscopic loading.

Conclusively, under both drained and undrained conditions in the vascular pores, the osteocytes experience, irrespective of the uniaxial or hydrostatic nature of the macroscopic strain applied at physiologically relevant magnitudes and frequencies, hydrostatic pressures of a magnitude which has been shown to be very effective in stimulating various biological cells, including osteocytes (compare Table 2), see also Klein-Nulend et al. (2012). Moreover, once the vascular pores become undrained, they become pressurized at the same level as the one experienced by the lacunar pores, i.e. the one triggering cellular activity.

### Development of lacunar pore pressure due to bone aging

The presented model can also be utilized to study the effect of degenerative diseases, such as osteoporosis, on the mechanical stimulation of osteocytes. In this context, the lacunar pores get increasingly mineralized over time, thus the lacunar porosity decreases. Assuming that the number of lacunar pores reaches a maximum at an age of 18 years, with a volume fraction of $\bar{f}_\text {lac}^\text {initial}=0.1$, the histomorphometric study implemented by Busse et al. (2010) suggests a lacunar pore volume fraction rate of $\text{ d }\bar{f}_\text {lac}/\text{ d }t\approx -5\times 10^{-4}\,\text {y}^{-1}$. The vascular porosity, in turn, increases significantly with increasing age. This increase was quantified by Cooper et al. (2007), by means of a comprehensive analysis of microcomputed tomography images gained from the femoral midshafts of subjects aged between 18 and 92 years. Cooper et al. (2007) show that the inverse of the cortical vascular porosity may decrease linearly, with $\text{ d }[(f_\text {vas})^{-1}]/\text{ d }t\approx -0.42\,\text {years}^{-1}$, thus the corresponding porosity increase follows an exponential trend. These porosity evolutions were entered into our poromicromechanical model, in order to elucidate the corresponding lacunar pore pressure changes. For the loading boundary condition, we consider a body weight of 75 kg, and an area of the cortex in the adult femoral midshaft of 60 mm$^2$ (Gosman et al. 2013). For getting an estimate for the stresses possibly arising in the cortex, we furthermore assume a moderate loading, amounting to $\sigma _{\text {macro},33}\approx -500$ kPa (when taking aforementioned numbers as reference).

**Fig. 7:**
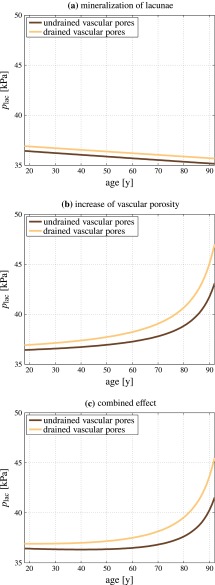
Lacunar pore pressure evolution during bone aging: **a** the effect of decreasing lacunar porosity, **b** the effect of increasing vascular porosity, and **c** combination of **a** and **b**; all computations are performed for undrained and drained vascular pores

Our model suggests that the lacunar pressure decreases over time, when neglecting the effect of increasing vascular porosity, see Fig. 7a. This would mean that additionally to a decreasing number of osteocytes which could sense the mechanical loading and changes thereof, the osteocytes would also sense less of the applied mechanical loading. While such trend is observed irrespective of whether the vascular pores are drained or undrained, in absolute numbers drained vascular pores induce slightly higher lacunar pore pressures. However, when neglecting the decreasing lacunar porosity and considering only the vascular porosity increase, we observe a significant increase in the arriving stress stimulus over time for a constant mechanical loading, see Fig. 7b. Thus, the osteocytes would be confronted with higher pore pressures that they transduce into corresponding biochemical processes. Again, the lacunar pore pressure for drained vascular pores would be higher than the pressure for undrained vascular pores. When combining both effects, see Fig. 7c, we observe a long-term increase in the lacunar pore pressures, whereas up to an age of $\approx$ 50 years the until then slightly dominating effect of lacunar porosity decrease leads to an actually reduced pore pressure. However, this effect is of minor magnitude, and hardly visible in Fig. 7c.

We note that these results do not contradict the widespread notion of “old” bone being less responsive to changes of the mechanical loading than ”young” bone (Duncan and Turner 1995; Turner et al. 1995). Figure 7 merely suggests that the pore pressure to which osteocytes are subjected increases with increasing age. The net response of the bone metabolism to changes of the mechanical loading, however, is not only governed by the magnitude of the mechanical stimulus, but also by the osteocyte density, viability, and connectivity. The latter quantities decrease with age, as is implied by the aging-related decrease of the lacunar porosity (Busse et al. 2010). Furthermore, in the elderly, aging-related behavioral changes of the involved cells represent an additional key factor, contributing to the reduced ability of cells to appropriately translate mechanical stimuli into corresponding bone remodeling responses, see e.g., Klein-Nulend et al. (2002), Pearson and Lieberman (2004), Leppänen et al. (2008) and Onal et al. (2013).

## Discussion

Motivated by the work of Gardinier et al. (2010), which strongly suggested the occurrence of poromechanically undrained lacunar pores during physiological loading cycles, as explained in more detail in Sect. 1, we here used a well-validated poromicromechanical model to predict undrained lacunar pore pressures under physiological loading conditions, and the obtained pressures beautifully match those which have been experimentally shown to stimulate a variety of cells, including bone cells (see Table 2). In this context, it is most important to clearly note that such hydrostatic pressures at the lacunar pore level are triggered by strongly non-hydrostatic macroscopic stress states acting on a piece of cortical or trabecular bone.

### Poromicromechanical modeling of bone: current limitations and potential extensions

Our current analysis aims at a reasonable and reliable estimation of the lacunar hydrostatic pressure, based on classical poroelasticity theory, and taking into account that bone is a hierarchically organized, multiporous material. Employing the concept of continuum micromechanics, the composition and morphology of (cortical or trabecular) bone is considered in terms of poromechanically governing microstructural features, such as pore shapes and porosities, as well as elastic stiffnesses of the solid phases. This way, it was shown that macroscopic loading under normal physiological conditions leads to the buildup of hydrostatic pressures in undrained lacunar pore spaces. These pressures are high enough for effectively stimulating the osteocytes residing in the lacunar pores (see the previous section). However, the present contribution leaves aside a Darcy-type transport model, which, in combination with the poromechanics model presented in this paper, would also allow for simulation of load-induced fluid transport and consolidation processes. Such transport models and estimation of respective permeabilities have been intensively discussed for the lacunar-canalicular fluid flow problem, see, e.g., Weinbaum et al. (1994), Cowin et al. (2009) and Nguyen et al. (2010).

A straightforward extension of our model, then allowing for fluid flow computations, would concern the formulation of a Darcy-type relation between the lacunar pressure gradient defined on the scale of the extravascular RVE, and a corresponding fluid flow. The underlying permeability of the lacunae-canaliculi network could be chosen based on data available in literature, see, e.g., Cardoso et al. (2013). Alternatively, a more elegant and elaborate approach could be taken, involving the explicit introduction of the canaliculi as cylindrical pores within the extracellular bone matrix. When considering the flow in the canaliculi, in a first approximation, as of the Poiseuille type, then this flow can be straightforwardly upscaled, so as to arrive at the homogenized permeability of the extralacunar matrix. Corresponding mathematical expressions have been recently derived, but used at another scale, namely at that of trabecular bone with “vascular canals” (Abdalrahman et al. 2015). At an even more elaborate stage, transport modeling may be extended to more than one pore space, in particular to the exchange of fluid between the lacunar-canalicular and the vascular pore space, as has been proposed by Cowin et al. (2009). In more detail, the Biot coefficient and moduli reported in this paper could well be fed by values emanating from the double-porous model which we have described in the present paper; while the permeability tensor could be derived from the aforementioned model of Abdalrahman et al. (2015). In a further sophistication step of the model, the flow pattern in the canaliculi could be resolved beyond Poiseuille’s assumption of a parabolic velocity profile, so as to distinguish between the cell processes located at the center of the cross sections through the canaliculi, and the surrounding pericellular matrix potentially comprising also some tethering elements (You et al. 2004; Thompson et al. 2011; Wang et al. 2014).

### Fluid flow and undrained hydrostatic pressure as periodically alternating mechanical stimuli of bone cells; and their relations to transport phenomena

The instances of undrained conditions are typically expected around maximum loading rates, while states in-between may well allow for fluid flow within the canaliculi, and these fluid flow cycles may express themselves in terms of cycles of so-called streaming potentials; such potential electrokinetic effects have been proposed by Eriksson (1974), Pienkowski and Pollack (1983), Pollack et al. (1984) and Weinbaum et al. (1994). In turn, the aforementioned cyclic electrical potentials do not necessarily imply transport of fluid, as bone is also known to be piezoelectric (Fukada and Yasuda 1957; Bassett 1968; Marino and Becker 1970; Marino et al. 1971; Reinish and Nowick 1975; Zhang et al. 2012)—and distinction between piezoelectric and electrokinetic effects turns out to be difficult, not to say impossible (Ahn and Grodzinsky 2009). Coincidently, piezoelectricity is sometimes regarded as important factor for biological responses (Ferreira et al. 2009; Fernández et al. 2012).

Other experiments which have been employed for arguing in favor of significant canalicular fluid flow, concern tracer molecules which were injected into bone specimens. Then, histological studies were carried out in bone cross sections with and without additional mechanical loading applied, e.g., through four point-bending protocols (Knothe Tate et al. 2000). It turned out that mechanical loading enhanced the tracer transport, up to 30 % when estimated from a threshold-based voxel analysis of photomicrographs—see Knothe Tate and Knothe (2000) for details, especially Table 2 of this reference—and by not more than 5 % when quantified through a confocal microscopy-based study—see Figure 4 of Knothe Tate and Knothe (2000).

The popular explanation for transport of tracer molecules, which has been advocated more and more explicitly over time (Seliger 1970; Knothe Tate and Knothe 2000; Knothe Tate et al. 2000; Wang et al. 2005; Zhou et al. 2008; Kwon and Frangos 2010; Price et al. 2011; Kwon et al. 2012), is that without mechanical loading, only diffusive transport is taking place, while mechanical loading might induce an additional convective transport portion, thus enhancing the overall transport of tracer through the investigated bone tissue. In order to further discuss this popular explanation, it is quite instructive to more closely examine the load cases which were actually applied to the system studied by Knothe Tate and Knothe (2000): Their strain gauge recordings show that loading pulses lasting around 2 s are intermitted by 13-s-long quiescent periods where no (macroscopic) strain was measured at all. Since absence of such strain provokes neither undrained hydrostatic pressure nor pore fluid flow, we restrict our analysis of the applied load cycles to the 2 s-intervals where actual strains were recorded. The results are summarized in Table 3: the most frequently occurring characteristic times range from 0.1 to 1 s, longer times cover only a few percent of the entity of such times, and not a single one of the characteristic times exceeds 10 s. This, together with the arguments given in the Introduction (see Sect. 1), qualifies fluid flow as a kind of secondary phenomenon taking place in the study of Knothe Tate and Knothe (2000), provoking the obvious question what drives then the enhanced tracer transport under oscillatory loading.

**Table 3:** Distribution of characteristic loading times related to the ex vivo compression tests on the forelimb of the Swiss alpine sheep, in particular on the compact part of the metacarpus therein, conducted by Knothe Tate and Knothe (2000); I: $T_\text {load}<0.1\,$s, II: $0.1\,\text {s}\le T_\text {load}< 1\,$s, III: $1\,\text {s}\le T_\text {load}< 10\,$s, and IV: $10\,\text {s}\le T_\text {load}$

Load cycle	I (%)	II (%)	III (%)	IV (%)
1	8	65	27	0
2	11	79	10	0
3	13	84	3	0
4	13	87	0	0
5	12	70	18	0
6	10	63	27	0
7	8	78	14	0

Seeking such an explanation, we recall some fundamental principles of physical chemistry: When polarized fluids, such as water, are adjacent to charged surfaces, such as the experimentally observed mineral-rich surfaces of extracellular bone tissue (Lees et al. 1984; Lees and Prostak 1988; Lees et al. 1994; Sasaki et al. 2002), the water molecules become ordered, leading to a substance called “layered” or “structured” water (Pollack 2001, 2013), which exhibits increased viscosity and decreased diffusivity (Ichikawa et al. 2002; Pivonka et al. 2004), when compared to fully disordered “bulk water”. The zone of layered water, which is also called exclusion zone (Pollack 2013), may reach thicknesses of up to several milimeters (Florea et al. 2014), which recently allowed for explaining the permeability properties of trabecular bone (Abdalrahman et al. 2015). Remarkably, for a variety of biological material systems (Green and Otori 1970; Wilson and Dietschy 1974; Barry and Diamond 1984; Pollack and Clegg 2008) it has been shown that stirring of layered fluids led to a diffusivity re-gain. Thus, mechanical excitation of the bone fluid in form of oscillating hydrostatic pressures resulting from macroscopic loading might result in partial “destruction” of the water layering effect, and therefore explain the transient acceleration of tracer transport under oscillating mechanical loading. Once the diffusion process is completed, however, no difference between loaded and unloaded configurations would be expected, as was indeed found experimentally by Knothe Tate and Knothe (2000).

### Summary and outlook

Conclusively, no major experimental findings necessarily contradict the potential occurrence of undrained lacunar pore pressure occurring during physiological load cycles. Then, given the novel poromicromechanical results presented in the present paper together with all the experimental evidence condensed into Table 2, this pressure appears as a prime candidate for being used as a mechanical stimulus in the context of multiscale mechanobiological approaches linking poromicromechanics and mathematical systems biology (Scheiner et al. 2013, 2014).

## Appendix: Definition of poroelastic quantities

### Upscaling (drained) stiffness, Biot tensors, and Biot moduli

We start with considering the constituents of the extravascular RVE (see Fig. 3), i.e., the extralacunar matrix and the lacunar pores, as poroelastic materials, with constitutive laws reading as

23 $$\begin{aligned} \varvec{\sigma }_i = \mathbb {C}_i : \varvec{\varepsilon }_i - \mathbf {b}_i p_i\,,\quad i\in \{\text {exlac},\text {lac}\}\,, \end{aligned}$$

where $\varvec{\sigma }_i$ is the stress tensor of constituent *i*, $\varvec{\varepsilon }_i$ is the strain tensor of constituent *i*, $\mathbb {C}_i$ is the drained stiffness tensor of constituent *i*, $\mathbf {b}_i$ is the Biot tensor of constituent *i*, and $p_i$ is the pressure to which constituent *i* is exposed. More specifically, the extralacunar bone matrix is considered as purely solid phase, so that

24 $$\begin{aligned} \mathbf {b}_\text {exlac}=0 \Rightarrow \varvec{\sigma }_\text {exlac} = \mathbb {C}_\text {exlac}:\varvec{\varepsilon }_\text {exlac}\,, \end{aligned}$$

while the lacunar pores do not exhibit any solid stiffness, and are just subjected to a pore pressure, so that

25 $$\begin{aligned} \mathbf {b}_\text {lac}= \mathbf {1} \Rightarrow \varvec{\sigma }_\text {lac} = -\mathbf {1}p_\text {lac} \end{aligned}$$

where $\mathbf {1}$ denotes the second-order unit tensor. The corresponding “homogenized” material behavior of the extravascular bone material follows the standard relation of continuum micromechanics (Zaoui 2002; Dormieux et al. 2006; Hellmich et al. 2009):

26 $$\begin{aligned} \varvec{\sigma }_\text {exvas}= \mathbb {C}_\text {exvas}:\varvec{\varepsilon }_\text {exvas}-\mathbf {b}_\text {exvas}^\text {lac}p_\text {lac}\,, \end{aligned}$$

with

27 $$\begin{aligned} \mathbb {C}_\text {exvas}= \bar{f}_\text {exlac}\mathbb {C}_\text {exlac}:\mathbb {A}_\text {exlac} \end{aligned}$$

and

28 $$\begin{aligned} \mathbf {b}_\text {exvas}^\text {lac}= \bar{f}_\text {lac}\mathbf {1}:\mathbb {A}_\text {lac}\,, \end{aligned}$$

whereby $\bar{f}_\text {lac}$ and $\bar{f}_\text {exlac}$ are the volume fractions of the lacunar pore space and the extralacunar space, quantified within the RVE of extravascular bone matrix, compare Fig. 3. Furthermore, $\mathbb {A}_\text {exlac}$ and $\mathbb {A}_\text {lac}$ are the concentration (or “downscaling”) tensors relating extravascular strains to those in the extralacunar and (drained) lacunar spaces,

29 $$\begin{aligned} \varvec{\varepsilon }_i= \mathbb {A}_i : \varvec{\varepsilon }_\text {exvas}\,,\quad i\in \{\text {lac},\text {exlac}\}\,. \end{aligned}$$

These concentration tensors quantify the micromechanical interactions between a transversely isotropic extralacunar matrix of stiffness $\mathbb {C}_\text {exlac}$, and spherical (drained) lacunar pores. They are derived from combination of matrix-influence problems of the Eshelby-Laws type (Eshelby 1957; Laws 1977), with the (micro) strain average rule valid for the extravascular RVE. The corresponding dependence of $\mathbb {A}_\text {exlac}$ and $\mathbb {A}_\text {lac}$ on $\bar{f}_\text {lac}$ and $\mathbb {C}_\text {exlac}$ is given in great mathematical detail in Morin and Hellmich (2014), where the extralacunar space is called “extracellular space”. The stiffness of the latter space is a function of its mass density only, due to the existence of experimentally corroborated “universal” bone composition rules between organic, mineral, and water contents (Vuong and Hellmich 2011), as well as of fibrillar organization rules depending on the mineralization and hydration degrees only (Hellmich and Ulm 2003; Morin and Hellmich 2013; Morin et al. 2013). These rules hold irrespective of species, age, or anatomical location, as long as the organism is not drug-treated. Accordingly, an extracellular mass density of 1.93 g/cm$^3$, typical for human femur (Lees et al. 1983), results, according to the very comprehensively experimentally validated microelasticity theory as summarized in (Morin and Hellmich 2014), in an extralacunar stiffness of

30 $$\begin{aligned} \mathbb {C}_\text {exlac}= \left( \begin{array}{c@{\quad }c@{\quad }c@{\quad }c@{\quad }c@{\quad }c} 22.88 &{} 8.93 &{} 10.14 &{} 0 &{} 0 &{} 0 \\ 8.93 &{} 22.88 &{} 10.14 &{} 0 &{} 0 &{} 0 \\ 10.14 &{} 10.14 &{} 29.60 &{} 0 &{} 0 &{} 0 \\ 0 &{} 0 &{} 0 &{} 14.72 &{} 0 &{} 0 \\ 0 &{} 0 &{} 0 &{} 0 &{} 14.72 &{} 0 \\ 0 &{} 0 &{} 0 &{} 0 &{} 0 &{} 13.96 \\ \end{array}\right) \,\text {GPa}\,. \end{aligned}$$

We take this stiffness tensor as the basis for all computations given in the present paper—noting that it is conveniently close to the famous direct measurements on human femur performed by Ashman et al. (1984).

One hierarchical level further up, i.e., within an RVE of macroscopic bone material (see Fig. 3), the extravascular bone matrix plays the role of a (poroelastic) phase with state equation (26), the other phase being the vascular pores, characterized by

31 $$\begin{aligned} \mathbf {b}_\text {vas}= \mathbf {1} \Rightarrow \varvec{\sigma }_\text {vas}= -\mathbf {1}p_\text {vas}\,. \end{aligned}$$

Applying homogenization rules analogous to those having led to Eqs. (26)–(28), we upscale Eqs. (26) and (31) one level up, so as to arrive at Eq. (2), whereby

32 $$\begin{aligned} \mathbf {b}_\text {macro}^\text {lac}=\bar{f}_\text {exvas} \mathbf {b}_\text {exvas}^\text {lac}:\mathbb {A}_\text {exvas}\,, \end{aligned}$$

and

33 $$\begin{aligned} \mathbf {b}_\text {macro}^\text {vas}=\bar{f}_\text {vas} \mathbf {1}:\mathbb {A}_\text {vas}\,, \end{aligned}$$

with $\mathbb {A}_\text {exvas}$ and $\mathbb {A}_\text {vas}$ as the concentration tensors downscaling strains from the macroscopic to the extravascular and (drained) vascular spaces,

34 $$\begin{aligned} \varvec{\varepsilon }_i= \mathbb {A}_i:\mathbf {E}\,,\quad i\in \{\text {vas},\text {exvas}\}\,. \end{aligned}$$

Mathematical details on these concentration tensors can again be found in (Morin and Hellmich 2014).

The Biot moduli occurring in the state equations for the porosity changes, Eqs. (3) and (4), can be derived from the superposition of pore pressure and (macro) strain-related load cases in conjunction with the stress average rule (Dormieux et al. 2006; Hellmich et al. 2009). In case of the extravascular space, we arrive at

35 $$\begin{aligned} \frac{1}{N_\text {exvas}}= -\mathbf {b}_\text {lac}:\left( \mathbb {C}_\text {excell}\right) ^{-1}: \left( \bar{f}_\text {lac}\mathbf {b}_\text {lac}- \mathbf {b}_\text {exvas}^\text {lac} \right) \,, \end{aligned}$$

which then enters the following expressions for the Biot moduli on the macroscopic scale

36 $$\begin{aligned} \frac{1}{N_\text {macro}^\text {lac,lac}}=&\, -\mathbf {b}_\text {exvas}^\text {lac}: \left( \mathbb {C}_\text {exvas} \right) ^{-1}\nonumber \\&\,: \left( f_\text {exvas}\mathbf {b}_\text {exvas}^\text {lac}- \mathbf {b}_\text {macro}^\text {lac}\right) +\frac{f_\text {exvas}}{N_\text {exvas}}\,,\end{aligned}$$

37 $$\begin{aligned} \frac{1}{N_\text {macro}^\text {vas,vas}}=&\, -\mathbf {b}_\text {exvas}^\text {vas}: \left( \mathbb {C}_\text {exvas} \right) ^{-1}: \left( f_\text {vas}\mathbf {b}_\text {vas}- \mathbf {b}_\text {macro}^\text {vas}\right) \,,\end{aligned}$$

38 $$\begin{aligned} \frac{1}{N_\text {macro}^\text {lac,vas}}=&\, -\mathbf {b}_\text {exvas}^\text {lac}: \left( \mathbb {C}_\text {exvas} \right) ^{-1}:\left( f_\text {vas} \mathbf {b}_\text {vas}-\mathbf {b}_\text {macro}^\text {vas}\right) \,. \end{aligned}$$

### Elastic energy stored in the macroscopic RVE of bone

In the case of undrained lacunar and drained vascular pores, the elastic energy stored in a macroscopic RVE of bone amounts to

39 $$\begin{aligned} \varPsi ^\text {lac-u}= \frac{1}{2}\mathbf {E}:\mathbb {C}_\text {macro}^\text {lac-u}:\mathbf {E}\,, \end{aligned}$$

with $\mathbb {C}_\text {macro}^\text {lac-u}$ following Eq. (9). For uniaxial strain $\mathbf {E}=E_{ii}\mathbf {e}_i\otimes \mathbf {e}_i$, $i=1,2,3$, Eq. (39) reduces to

40 $$\begin{aligned} \varPsi ^\text {lac-u}= \frac{1}{2}C_{\text {macro},iiii}^\text {lac-u}(E_{ii})^2\,. \end{aligned}$$

For hydrostatic strain, $\mathbf {E}=E_\text {hyd}\sum _{i=1}^3\mathbf {e}_i\otimes \mathbf {e}_i$, Eq. (39) reduces to

41 $$\begin{aligned} \varPsi ^\text {lac-u}= \frac{1}{2}(E_\text {hyd})^2 {\sum \limits _{i=1}^3\sum \limits _{j=1}^3 C_{\text {macro},iijj}^\text {lac-u}}\,. \end{aligned}$$

In the case of both lacunar and vascular pores being undrained, the elastic energy stored in a macroscopic RVE of bone amounts to

42 $$\begin{aligned} \varPsi ^\text {lac,vas-u}= \frac{1}{2}\mathbf {E}:\mathbb {C}_\text {macro}^\text {lac,vas-u}:\mathbf {E}\,, \end{aligned}$$

with $\mathbb {C}_\text {macro}^\text {lac,vas-u}$ following Eq. (17). For uniaxial strain $\mathbf {E}=E_{ii}\mathbf {e}_i\otimes \mathbf {e}_i$, $i=1,2,3$, Eq. (42) reduces to

43 $$\begin{aligned} \varPsi ^\text {lac,vas-u}= \frac{1}{2}C_{\text {macro},iiii}^\text {lac,vas-u}(E_{ii})^2\,. \end{aligned}$$

For hydrostatic strain, $\mathbf {E}=E_\text {hyd}\sum _{i=1}^3\mathbf {e}_i\otimes \mathbf {e}_i$, Eq. (42) reduces to

44 $$\begin{aligned} \varPsi ^\text {lac,vas-u}= \frac{1}{2}(E_\text {hyd})^2 {\sum \limits _{i=1}^3\sum \limits _{j=1}^3 C_{\text {macro},iijj}^\text {lac,vas-u}}\,. \end{aligned}$$
